# Virulence between ESBL and non-ESBL producing isolates of Acinetobacter baumanii causing diabetic foot infections

**DOI:** 10.6026/973206300212942

**Published:** 2025-08-31

**Authors:** Hirdesh Kumari Gupta, Ramanath Karicheri, Abhishek Mehta

**Affiliations:** 1 Department of Microbiology, Index Medical College and Research Centre, Nemawar, Indore, Madhya Pradesh, India; 2 Department of Microbiology, Government Medical College Datia, Madhya Pradesh, India

**Keywords:** *Acinetobacter baumannii*, diabetic foot ulcer, gelatinase, multidrug resistance, pellicle formation

## Abstract

*Acinetobacter baumannii* is an opportunistic pathogen associated commonly with healthcare-associated-infections and predominantly involved in diabetic foot infections with virulence factors determining pathogenicity especially in multidrug resistant strains. Therefore, it is of interest to compare virulence factors (pellicle formation and gelatinase activity) between Extended-spectrum beta-lactamase (ESBL) and non-ESBL producing *Acinetobacter baumannii* isolates derived from diabetic foot ulcer infection (DFI). The isolates were identified using standard microbiological methods and antimicrobial susceptibility test was done by Kirby Bauer disk diffusion method. The double disk synergy test method (DDST) was used to detect ESBL production. The formation of pellicle at 37°C and gelatinase production indicated high virulence in ESBL producers. Colistin and polymyxin B were found to be the only options effective against multidrug-resistant *Acinetobacter baumannii* infections.

## Background:

*Acinetobacter baumannii* is an opportunistic pathogen associated with healthcare-associated-infections. It is gram-negative, coccobacilli, non-motile, non-fermentative, catalase-positive, oxidase-negative *aero*bic bacterium. These pathogens affect the people with a weak immune system. A.baumanii is the major cause of infections such as urinary tract infection (UTI), bacteremia, meningitis, pneumonia and burn wound infections. In the last decade, Acinetobacter strains have become resistant to most antibiotics due to irrational use of antibiotics, poor hygiene and prolonged hospital stay. The second most commonly found non- fermenter bacterial pathogen in Diabetic foot infections (DFIs) is *A. baumannii* [1, 2]. A few recognized virulence factors of *A.baumanni*i are biofilm, pellicle formation, siderophore, gelatinase production and cell surface hydrophobicity. Gelatinase is a protease enzyme produced by bacteria in ulcers/wounds that leads to the break-down of the collagen in subcutaneous tissue. Casein, haemoglobin and other bioactive peptides are also involved in inflammation and contribute to virulence [3]. Pellicle formation in *A. baumannii* allows them to survive on less nutrient-limited surfaces for several days [4]. The pellicle formation needs different proteins that are associated with pili formation, such as chaperone-usher system pili and the putative type III pili [5]. Various extracellular polymers are present in the matrix of *A.baumanni*i pellicles, like exopolysaccharides, lipopolysaccharides, which, along with cell surface appendages, contribute to the existence and persistence of *A. baumannii* in the clinical environment, exhibiting different cell properties in wounds [6]. In the environment of a hospital ward, pellicles occur in the form of minute droplets, which may be involved in bacterial colonization of diabetic foot ulcer patients who are already immunodeficient. The bacterial bunch may be separated from the pellicle and free to cause infection [7]. Therefore, it is of interest to evaluate and compare the virulence factors like pellicle formation and gelatinase activity between ESBL and non-ESBL-producing *A. baumannii* isolates from DFI.

## Materials and Methods:

This study is a cross-sectional study carried out in the Department of Microbiology, Index Medical College Indore after seeking approval from institutional ethics committee (Approval No. MU/research/EC/Ph.D/2023/348) The study was conducted according to the Declaration of Helsinki. A total of 54 non-duplicate *A. baumannii* isolates were obtained from clinical samples processed during 8 months from July 2024 to February 2025. Isolation and identification of test isolates were done using standard microbiological techniques. Antimicrobial susceptibility testing was done by Kirby-Bauer disk diffusion method and the interpretation was done as per CLSI 2024 guidelines [8].

## Pellicle or Air- liquid interface Biofilm (ALI) assay:

ALI assay or Pellicle assay was performed by inoculating 5 ml of Mueller-Hinton (MH) broth in two polystyrene tubes with a single colony of isolates of *A. baumannii* with an initial OD 600 of 0.01. For 3 days, the tubes were incubated at 25°C or 37°C without shaking. Pellicle formation appeared as a white layer on the surface of Muller Hinton broth. The positive control was used by *Pseudomonas aeruginosa* (PA01) and the negative control was used by *A. baumannii* ATCC 19606 and maintained simultaneously [5].

## Gelatinase assay:

The isolate was grown in brain heart infusion broth and incubated at 37°C for 18 h. One loopful of culture was inoculated onto Luria Bertani Agar containing gelatine (30 g/L). The plates were incubated overnight at 37°C and then cooled for 5 h at 4°C. The positive result was indicated by the appearance of a turbid halo around colonies on the medium for gelatinase production [3].

## Results:

Out of the total of 54 isolates of *A. baumannii* derived from the cases of Diabetic foot infection, half (27) turned out to be ESBL producers. All the patients had comorbidities with hypertension, nephropathy, neuropathy, retinopathy, peripheral vascular disease and type 2 diabetes mellitus. The Age of the patients ranged from 30 to 80 years and the majority of patients were between the ages of 44 and 64 years.

## Gelatinase production:

All the 54 isolates were multidrug resistant, with gelatinase production reported in 19 (35%), out of which 11 (58%) were ESBL producers, which were predominant gelatinase producers as compared to others.

## Pellicle formation:

A thin pellicle appeared at the surface of the broth 24 hours post-incubation, with increasing thickness over time. By the end of the third day, an opaque and solid structure covered the entire liquid surface. 54 isolates of *A.baumanni*i from diabetic foot ulcers were visually analysed for pellicle formation. 48.14% (13/27) of pellicle-forming isolates, turned out to be ESBL producers. Pellicle formation and gelatinase production amongst ESBL and non-ESBL producing *A.baumanni* DFI isolates have been depicted in Table 1 (see PDF) and Table 2 (see PDF).

## Discussion:

Patients of type 2 diabetes mellitus experience DFI during their lifetime with a male preponderance [9]. ESBL isolates were more virulent than non-ESBL ones. Production of ESBL and multidrug resistance by microbes and their infections is associated with the increased hospital stay, higher morbidity, mortality rate and cost burden to the patients. Though some generalised studies have reported gelatinase production in *A. baumannii* isolates, they had not specifically taken DFI cases into consideration. In our study, gelatinase production was reported in 40.74% of ESBL producers and 29.62% of non-ESBL isolates of *A.baumanii*, which is similar to the study done by Mehmood *et al.* [10] (38.09% and 28.57% respectively). In another study done by Valli *et al.* [11] Gelatinase production was observed amongst 60% of *A. baumannii* isolates. But Covahir *et al.* [12] reported gelatinase production in only 14% of cases. In our study, pellicle formation was observed among 54 DFU isolates of *A. baumannii*. 48.14% of ESBL producers and 33.3% of non-ESBL producers were forming a pellicle at 25°C. At 37°C, pellicle formation was reported in 59.25% of ESBL producers and 44.44% of non-ESBL ones, respectively. Similarly, Marti *et al.* [5] reported pellicle formation at 25°C and 37°C among 35.9% and 12.2% of test isolates, respectively. Chabane *et al.* [4] have reported pellicle formation at 25°C in 30.32% of *A.baumanni*i isolates. Sara *et al.* [13] reported that the *A. baumannii* group has a higher ability to form pellicles than other pathogens and this feature could be connected to the higher pathogenicity of *A. baumannii*. A similar study was conducted in Iran by Mirbag HH *et al.* wherein a total of 113 *K. pneumoniae* isolates including 56 ESBL and 57 non ESBL-producers were collected whose Enzymatic profile, hypermucoviscosity and biofilm formation were investigated phenotypically unlike our study which was restricted to the pellicle formation and gelatinase activity. In addition virulence determining genes like *rmpA*, *aerobactin*, *kfu*, *allS*, *mrkD*, *ybtS*, entB, *iutA*, fimH, wabG, wcaG, *K1* and K2 genes were detected through PCR (Polymersae chain reaction). The hypermocoviscosity was observed more often by non-ESBL producers as compared to the ESBL producers which exhibited a higher tendency towards Biofilm formation. Among the virulence genes, *K1*, *rmpA*, *iutA*, and *aero* were observed only in non-ESBLs [14].

## Conclusion:

Air-liquid interface biofilm/Pellicle formation and gelatinase production by DFI isolates of *A. baumannii* was reportedly high in ESBL producers as compared to non-ESBL producers. These virulence factors play a significant role in pathogenicity with in multidrug-resistant *A. baumannii* infections.

## Authorship:

All authors contributed significantly to the study and approved the final draft.

## Funding:

Nil
